# *Angiostrongylus vasorum* in wolves in Italy: prevalence and pathological findings

**DOI:** 10.1186/s13071-017-2307-1

**Published:** 2017-08-11

**Authors:** Claudio De Liberato, Goffredo Grifoni, Raniero Lorenzetti, Roberta Meoli, Cristiano Cocumelli, Antonio Mastromattei, Francesco Scholl, Pasquale Rombolà, Pietro Calderini, Gianpaolo Bruni, Claudia Eleni

**Affiliations:** Istituto Zooprofilattico Sperimentale del Lazio e della Toscana “M. Aleandri”, Via Appia Nuova 1411, 00178 Rome, Italy

**Keywords:** *Angiostrongylus vasorum*, *Canis lupus*, Wolf, Wildlife, Italy

## Abstract

**Background:**

*Angiostrongylus vasorum* is a nematode residing in the heart and pulmonary vessels of dogs and wild carnivores. In Europe the red fox is its reservoir, while only three records from wolves have been published. *Angiostrongylus vasorum* has a worldwide distribution, and many pieces of evidence demonstrate that it is spreading from endemic areas to new ones. In Italy, *A. vasorum* was reported with increasing frequency in dogs and foxes in the last decades, and now it is considered endemic throughout the country. *Angiostrongylus vasorum* can be asymptomatic or cause respiratory and circulatory disorders, at times causing severe disseminated infections.

**Methods:**

Between February 2012 and December 2016, 25 wolves found dead in central Italy were submitted to the Istituto Zooprofilattico del Lazio e della Toscana for post-mortem examination. Samples of lungs, heart, liver, spleen, kidneys, mediastinic lymph nodes and brain were collected from each animal for histological examination. When adult and larval nematodes were microscopically seen in lungs, the other organs were processed, and five histological sections for each organ were examined. To confirm parasite identification, lung samples were submitted to a PCR-sequencing protocol targeting the ITS2 region of *A. vasorum.*

**Results:**

Seven wolves (28.0%) harboured nematode larvae in lung sections. In two of the positive wolves, adult nematodes were visible in pulmonary arteries, in four animals larvae were also detected in other organs. DNA sequencing reactions confirmed parasite identification as *A. vasorum* in all the cases.

**Conclusions:**

As a result of the high prevalence of *A. vasorum* reported in wolves in the present study, a focus of high circulation could be hypothesised in central Italy. Nevertheless, the similarly high prevalence in foxes originating from the same areas were reported in previous papers. Histopathological evidence highlights the pathogenic potential of *A. vasorum* in the wolf, especially in juvenile animals.

## Background

Adult *Angiostrongylus vasorum* (Nematoda: Metastrongyloidea) reside in the heart and pulmonary vessels of dogs and many wild carnivore species [1–3]. Terrestrial gastropods, slugs and snails are the intermediate hosts [1]. In Europe, the red fox (*Vulpes vulpes*) is considered the natural reservoir of this parasite [1, 4, 5]. *Angiostrongylus vasorum* has a worldwide distribution, and many pieces of evidence point out that it is spreading from endemic areas to new ones [6–9]. In Italy, *A. vasorum* was first reported over 20 years ago in red foxes, and since then it has been reported with increasing frequency in dogs and foxes, and now it is considered endemic throughout the country [10, 11].

In the different host species, *A. vasorum* infection can be asymptomatic or cause respiratory and circulatory disorders [12, 13], at times causing disseminated infections of various severity [5, 14–16].

In the last few decades, the wolf (*Canis lupus*) has undergone a population recovery in Europe, thanks to legal protection and changes in human attitudes [17]. In Italy in particular, this recovery has been noticeable, and the wolf has expanded its distribution from small, isolated populations to the whole country, almost everywhere ecological conditions are suitable [18, 19]. Diseases are considered possible issues in the conservation of wild carnivores, particularly when spillover of pathogens between domestic and wild canids occurs [20, 21], but at present limited data have been published on the health status and parasite fauna of wolves in Europe and Italy.

At present, only three records of *A. vasorum* from wolves have been published [16, 22, 23]. Hence, in the above-described scenario, it was considered advisable to report new data regarding this parasite infection in this host, reporting a prevalence value and describing the more relevant histopathological findings.

## Methods

**Fig. 1:**
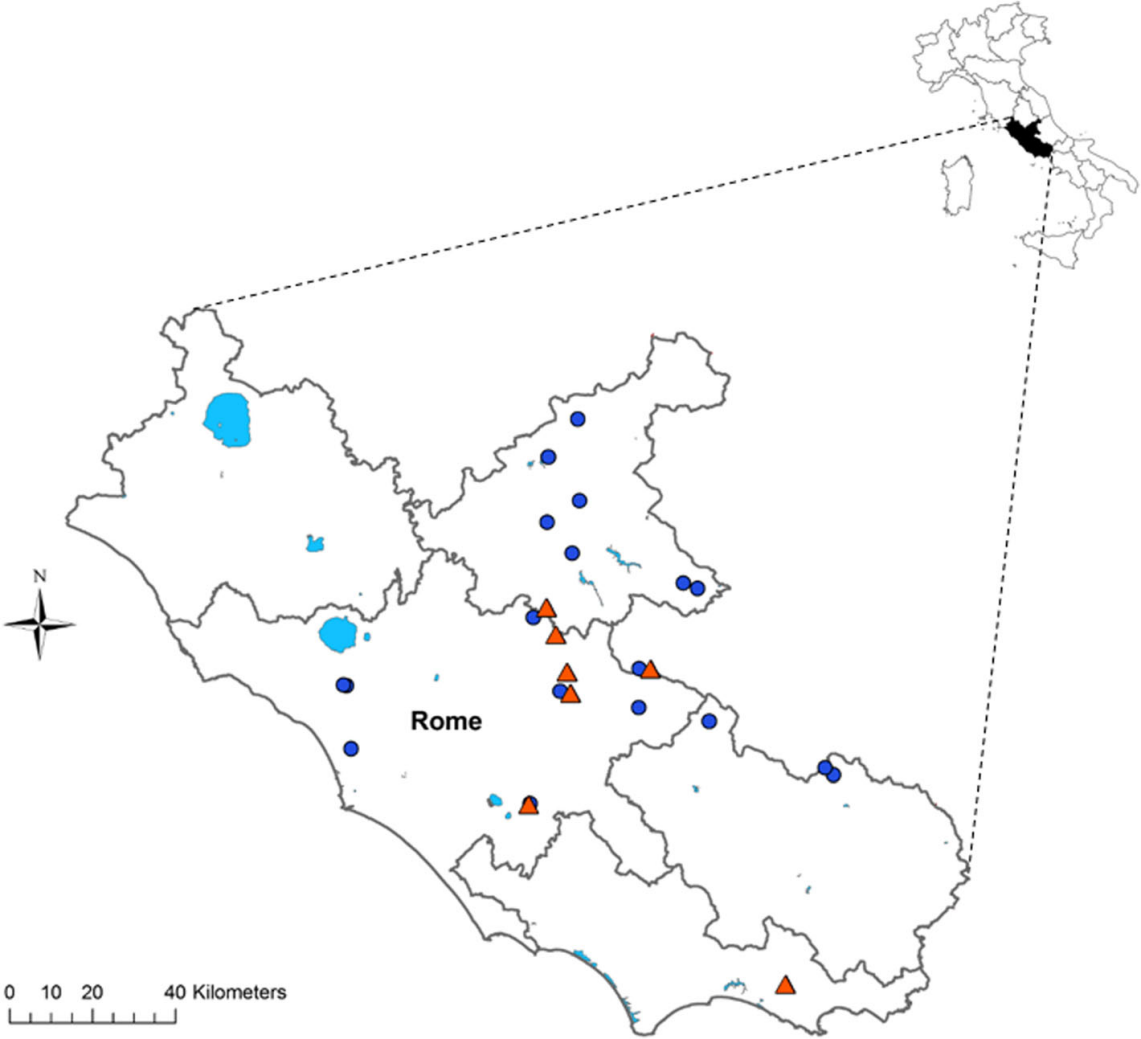
The study area in central Italy. Dots indicate negative wolves; triangles indicate wolves positive for *A. vasorum*

Between February 2012 and December 2016, 25 wolves (13 males and 12 females) found dead in Lazio Region (central Italy) (Fig. 1) were submitted to the Istituto Zooprofilattico Sperimentale del Lazio e della Toscana “M. Aleandri” for post-mortem examination. To ascertain if they were pure wolves or hybrids with the dog, 18 autosomal microsatellite markers were used to genotype each of the 25 animals [24]. The age of each animal was estimated trough teeth wear examination [25] in two classes, 10 being juveniles < 12 months and 15 adults > 12 months.

For histological exams, three samples of lungs and a sample of heart, liver, spleen, kidneys, mediastinic lymph nodes and brain were collected from each animal and fixed in buffered formalin. All lung samples were embedded in paraffin wax and 5 thin sections, stained with haematoxylin and eosin, were prepared for each sample and examined under a microscope. When adult and larval nematodes were microscopically seen in lung samples, the other organs previously fixed in formalin were processed as described above and 5 histological sections for each organ were examined with the aim of evaluating larval dissemination. To confirm parasite identification, lungs of all the wolves with adult nematodes or larval forms in lungs were subjected to a PCR-sequencing protocol, using the primers NC1 and NC2, as described by Gasser et al. [26]. Obtained sequences were compared with those available in the GenBank using the BLASTn tool.

## Results

**Fig. 2:**
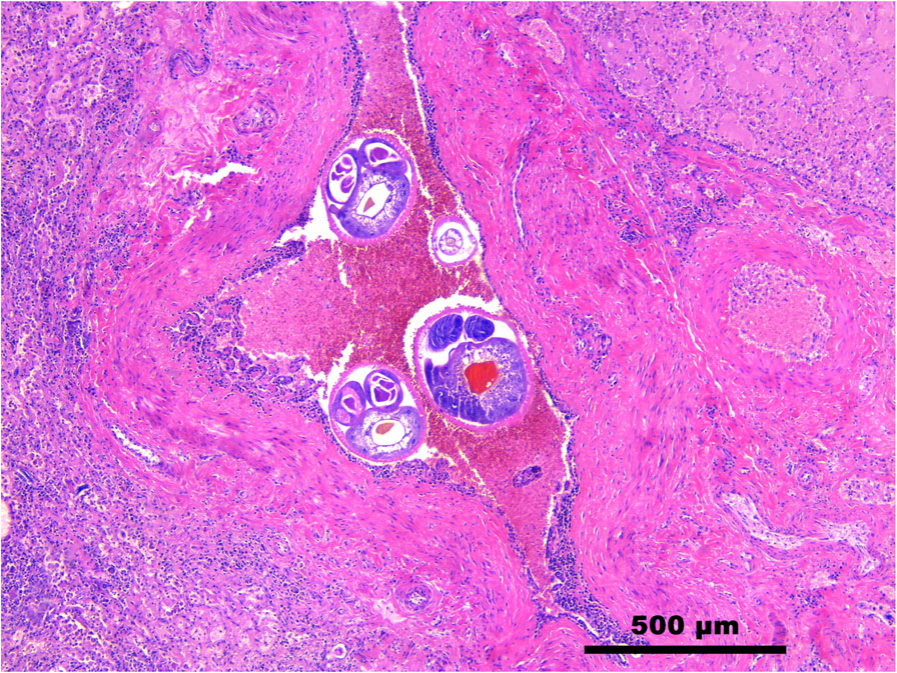
Wolf 4. Lung. Pulmonary artery containing adults of *A. vasorum*. Hematoxylin and Eosin staining

No genetic admixture with dogs for any of the 25 wolves was found. Seven wolves (28.0%) harboured nematode larvae in lung sections. In two of these, adult nematodes were visible in pulmonary arteries in lung sections (Fig. 2), and in 4 animals larvae were also detected in other organs (Table 1). Due to the previous report of *A. vasorum* in wolves from the same study area [16], anatomical localisation of adult worms in pulmonary vessels and finding of nematode larvae and histopathological changes in one of the examined organs led to the suspicion of an *A. vasorum* infection. DNA sequencing reactions, performed on 7 PCR positive samples, resulted in sequences roughly 480 bp in length. BLASTn analysis for all 7 sequences showed an identity of 99% (query coverage of 100%) with the ITS2 region of *A. vasorum* (GenBank: EU627597.1), confirming parasite identification (Table 1). Overall, *A. vasorum* prevalence was 38.5% among male specimens and 16.7% among female ones and 40.0% among juvenile wolves and 20.0% among adult ones.

**Table 1:** Age, sex and involved organs of the seven wolves positive for *Angiostrongylus vasorum*

			Adults	Larvae						
No.	Age^a^	Sex	Lungs	Lungs	Brain	Kidney	Liver	Heart	Lymph nodes	Lungs (PCR)
1	J	M	−	+	+	−	−	−	−	+
2	J	M	−	+	Flogosis	Flogosis	Flogosis	−	−	+
3	J	F	−	+	Flogosis	−	−	−	+	+
4	A	M	+	+	−	+	−	Flogosis	−	+
5	A	F	−	+	−	−	−	−	−	+
6	A	M	+	+	−	+	−	−	−	+
7	J	M	−	+	Flogosis	Flogosis	−	−	−	+

^a^A, > 12 months; J, < 12 months

At necropsy, all 7 positive wolves showed fatal traumatic lesions, with multiple bone fractures, and it was possible to ascertain that they all died due to fatal injuries. In Wolves 1, 4 and 6 a few congested and small consolidated areas were present in the pulmonary diaphragmatic lobes. Lungs of Wolf 2 showed several extensive areas of reddish-brown coloration with increased consistency in the diaphragmatic lobes and foci of pleural fibrosis with adhesions to the parietal pleura. In Wolf 3, only a whitish and firm pulmonary nodular lesion was seen. No gross changes were detected in lungs of wolves 5 and 7, as well as in any of the other organs of all seven wolves.

**Fig. 3:**
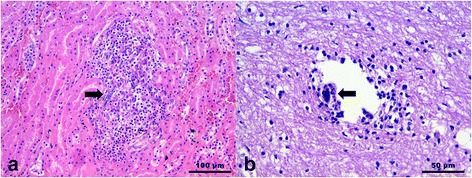
**a** Wolf 4. Kidney. *A. vasorum* larva (arrow) inside a cortical granulomatous focus. **b** Wolf 1. Brain. *A. vasorum* larva (arrow) in an encephalic vessel. Lymphocytes and plasma cells are surrounding the vessel. Hematoxylin and Eosin staining

Microscopically, parasitic pulmonary lesions were represented by small and scattered or confluent granulomatous foci, with lymphocytes, plasma cells, macrophages, granulocytes and a few multinucleated giant cells surrounding eggs or parasitic larvae. In Wolf 2, extensive areas of lymphoplasmacytic and eosinophilic pneumonia associated with necrosis and eggs and larval debris were seen. In Wolves 4 and 6, one or more adult worms were detected in pulmonary arteries (Fig. 2), frequently associated to organised thrombi. Larvae were also observed (Table 1) in 2 out of 7 kidneys, in glomerular capillaries or inside small cortical granulomas (Fig. 3a), in meningeal and encephalic vessels (Fig. 3b), in 1 out of 7 brains and in a subcapsular lymphatic sinus in 1 out of 7 mediastinic lymph nodes. In these cases, mild to moderate lymphoplasmacytic inflammation with few eosinophils accompanied the presence of the larvae. In some animals, despite no larvae were observed, small lymphoplasmacytic infiltrates with few eosinophils were seen in sections of different organs, brain and kidney in particular (Table 1). In the brain, mild to moderate flogosis was multifocally present in the leptomeninges and occasionally as perivascular cuffings in cerebral parenchyma. The kidney showed mild interstitial nephritis; inflammatory cells were sometimes located around the glomeruli or as small inflammatory nodules in the renal cortex.

## Discussion

When considering the lack of previous reports of *A. vasorum* in the wolf, the prevalence reported in the present study (28.0%) is surprisingly high. Two of the three previous reports of this host/parasite association were case reports [16, 22], not reporting prevalence values. The only previous prevalence reported in the literature is 3.1% [23] in Croatian wolves. Nevertheless, the two prevalence values are hardly comparable, due to the different techniques adopted. Hermosilla et al. [23] detected *A. vasorum* larvae from wolf faecal samples using the sodium acetate-acetic acid formalin technique and, as stated by the same Authors, this technique is not the most sensitive for the detection of lungworm larvae in faeces, thus their prevalence may be underestimated. The prevalence reported in the present study could be considered more realistic, due to the objectivity of recovering parasites at post mortem examination and the confirmation of their identification via molecular assays. Nevertheless, as adopted necropsy procedures were not specifically aimed at *A. vasorum* detection (i.e. pulmonary arteries were not systematically opened), false negatives can’t be ruled out. At present, *A. vasorum* in the wolf have been reported in a wide geographical range, from Spain on the west to Croatia to the east, indicating that the wolf should be considered one of the natural hosts of this nematode in Europe. Lack of reports from other areas are probably ascribable to the low wolf populations present in most of the geographical range of this species in Europe [27] and, in general, to the low number of wolves investigated for parasites.

As an alternative explanation for the high prevalence reported in the present study, a focus of high *A. vasorum* circulation should be hypothesised in the areas of central Italy from where the wolves originated. Nevertheless, Eleni et al. [11] reported a similarly high prevalence (43.5%) in foxes originating from the same areas, possibly indicating that natural areas of central Italy would be particularly favourable to this parasite. Moreover, considering the whole study area (Fig. 1), it is possible to pinpoint a cluster of positive animals in a restricted area northeast of Rome, an area probably characterised by environmental and climatic conditions (milder temperatures, higher humidity) particularly favourable to gastropods intermediate hosts of *A. vasorum* [28, 29].

Although low numbers did not allow any statistical analysis, interesting is the difference in prevalence recorded among adult and juvenile wolves and among female and male ones. In dogs higher levels of infection are recorded in young animals [7, 30], possibly due to an incomplete development of the immune system and to the inquisitive attitude of younger dogs, making more probable the ingestion of slugs or snails [12, 30]. Our results fit with these findings in dogs, with juvenile wolves showing a prevalence (40.0%) exactly twice as high compared to adults (20.0%). Regarding sex, we found a prevalence of *A. vasorum* in male wolves (38.5%) more than twice that in female ones (16.7%). In the literature, no gender predisposition to *A. vasorum* infection is reported [7] and among 20 dogs found infected in central Italy [31], exactly 50% were males and 50% females. In accordance with our results in wolves, only Chapman et al. [12] reported a higher percentage of male dogs (73.9%) among 23 animals found infected in England.

*Angiostrongylus vasorum* is known to be (at times) highly pathogenic in dogs [32] and to be able to cause significant pathology in foxes [11]. Hence, this parasite could be considered a potential health issue in regions where it is endemic, and wolves are of conservation concern. Histopathological evidence reported in the present study, consistent with the pathological findings previously reported in wolves [16], confirm the pathogenic potential of *A. vasorum* in this species, especially in juvenile animals, although the death of these wolves was ascribable to fatal injuries.
